# UNEQUAL LOSS: DISPARITIES IN RELATIONAL PROXIMITY TO A COVID-19 DEATH AMONG US OLDER ADULTS

**DOI:** 10.1093/geroni/igac059.1390

**Published:** 2022-12-20

**Authors:** Alicia Riley

**Affiliations:** University of California, Santa Cruz, Santa Cruz, California, United States

## Abstract

The COVID-19 pandemic has devastated communities of color in the U.S. at disproportionate rates. Racial-ethnic and language disparities in COVID grief may be even more extreme than those in individual mortality. We drew on the National Social Health and Aging Project (NSHAP) COVID Study, a supplement to the nationally-representative longitudinal survey. The analytic sample consisted of 2,554 community-dwelling older adults in the U.S. interviewed between September 2020 and January 2021. We used descriptive analysis to evaluate disparities by race/ethnicity/language subgroups in relational proximity to a COVID-19 death (aquaintaince < friend/family member < household member < spouse) and multiple logistic regression to evaluate disparities in experiencing at least one COVID-19 death in one’s social network. English-speaking, Non-Hispanic Black and Latino older adults were over-represented in every category of proximity to a COVID-19 death, but overrepresentation in proximity to a COVID-19 death was greatest among Spanish-speakers of any race. Although Spanish-speaking respondents were only 4.6% of the full sample, half of the respondents who lost a spouse to COVID-19 were Spanish-speakers. Disparities by language and race/ethnicity persisted even with the inclusion of controls for age, sex, marital status, education, and nativity. The most extreme disparities in closeness to COVID-19 death were experienced by Spanish-speakers of any race/ethnicity. It is unclear if this disparity is driven by language alone or the intersection of language and nativity and associated systemic vulnerabilities. Observed disparities could also reflect variation by language and immigrant identity in network size and structure, including connections to relatives in other countries.

